# Supplementary material for: Seismic arrival-time picking on distributed acoustic sensing data using semi-supervised learning
Source: Nat Commun. 2023 Dec 11;14:8192. doi: 10.1038/s41467-023-43355-3 (PMC10713581; doi:10.1038/s41467-023-43355-3)
Supplement: Supplementary file 1 — Supplementary Information [file 41467_2023_43355_MOESM1_ESM.pdf]

Supplementary Information

Seismic Arrival-time Picking on Distributed Acoustic Sensing Data using Semi-supervised Learning

Weiqiang Zhu[1,2], Ettore Biondi[1], Jiaxuan Li[1], Jiuxun Yin[1], Zachary E. Ross[1], and Zhongwen Zhan[1]

[1]California Institute of Technology

[2]University of California, Berkeley

Table S1: Comparison of picked and associated picks across different DAS arrays and models.

DAS array	Model	Total picks	Associated picks	Association ratio
Mammoth north	PhaseNet	4.2M	2.7M	0.64
	PhaseNet-DAS v1	14.3M	13.0M	0.91
	PhaseNet-DAS v2	35.9M	32.2M	0.90
Mammoth south	PhaseNet	5.3M	3.7M	0.69
	PhaseNet-DAS v1	18.8M	17.4M	0.92
	PhaseNet-DAS v2	40.2M	36.1M	0.90
Ridgecrest north	PhaseNet	1.1M	0.7M	0.67
	PhaseNet-DAS v1	7.2M	6.4M	0.89
	PhaseNet-DAS v2	11.9M	10.8M	0.93
Ridgecrest south	PhaseNet	13.2M	7.7M	0.59
	PhaseNet-DAS v1	28.5M	26.4M	0.93
	PhaseNet-DAS v2	48.0M	43.3M	0.90

Table S2: Parameters used for GaMMA phase association

Parameters	Values
Range	X: [-556.6, 556.6] km
	Y: [-556.6, 556.6] km
	Z: [0, 30] km
Velocity	P: 6.0 km/s
	S: 3.5 km/s
DBSCAN	eps: 30 s
	min samples: 1000
Covariance prior	1000
Oversampling factor	8
Min picks per event	500
Max time residual	1 s

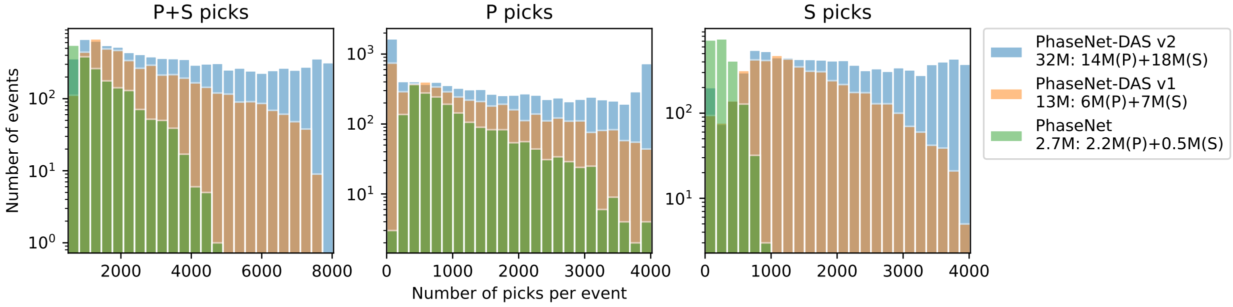

(a)

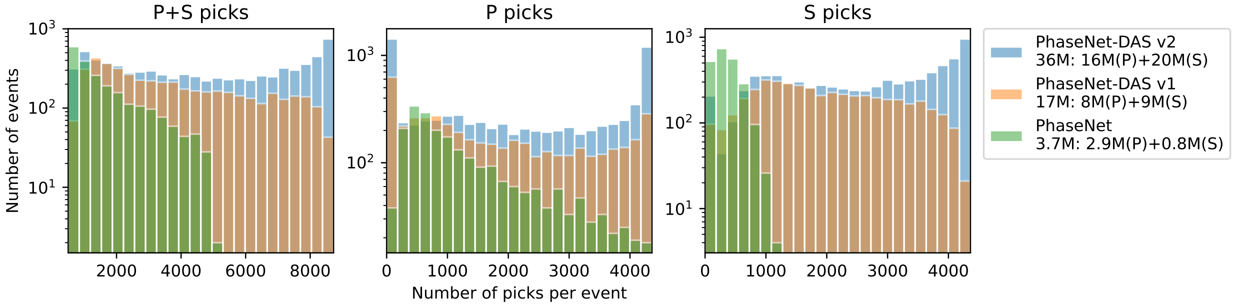

(b)

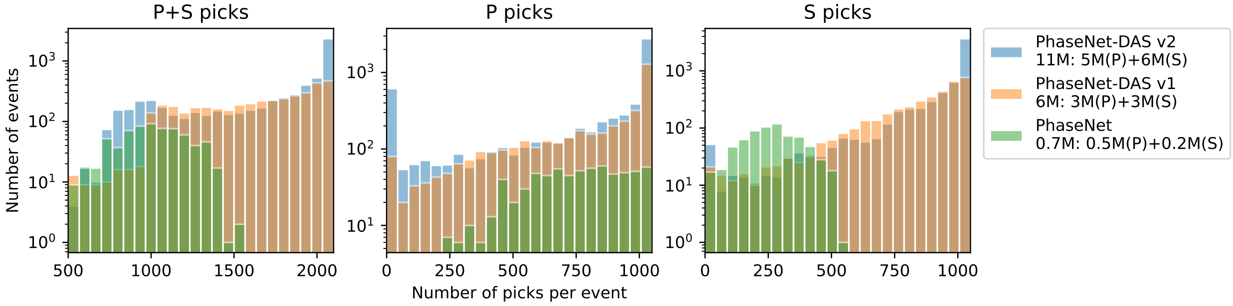

(c)

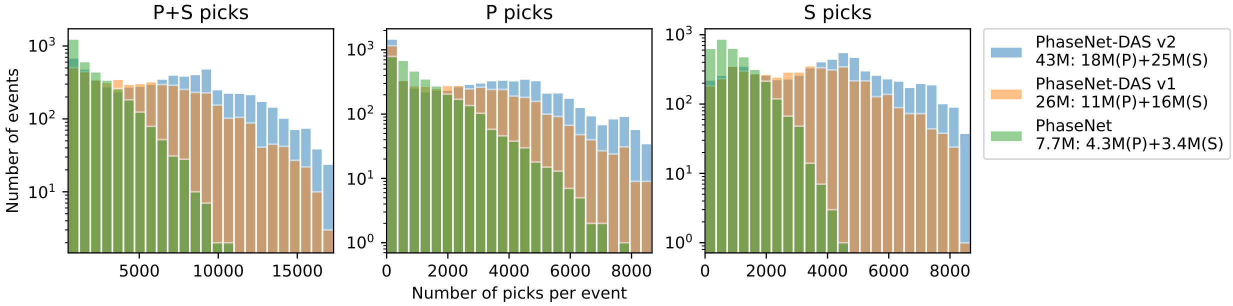

(d)

Fig. S1: Comparison of the number of P and S picks per event: (a) Mammoth north cable, (b) Mammoth south cable, (c) Ridgecrest north cable, and (d) Ridgecrest south cable.

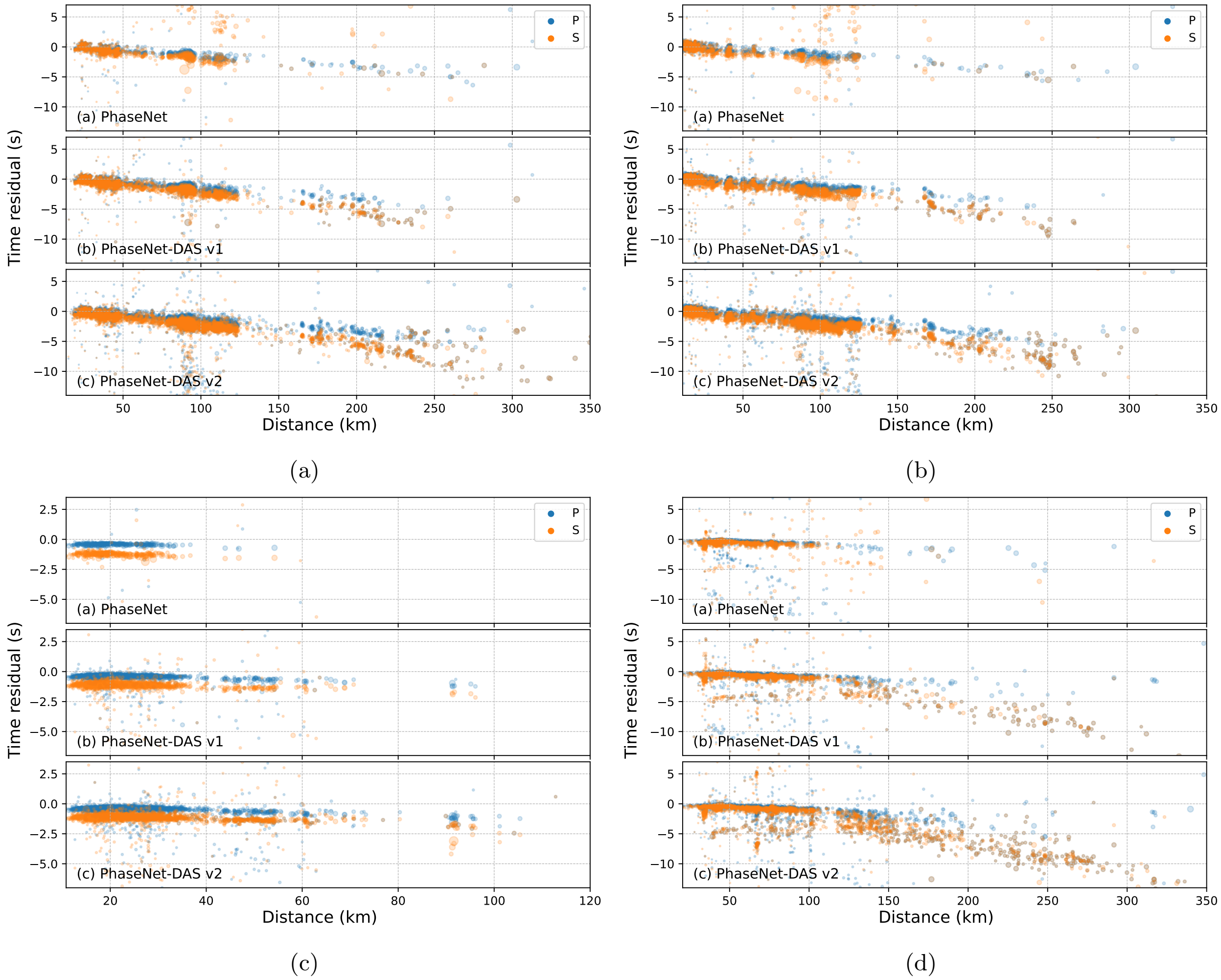

Fig. S2: Time residuals of P and S picks compared with theoretical arrival times: (a) Mammoth north cable, (b) Mammoth south cable, (c) Ridgecrest north cable, and (d) Ridgecrest south cable.

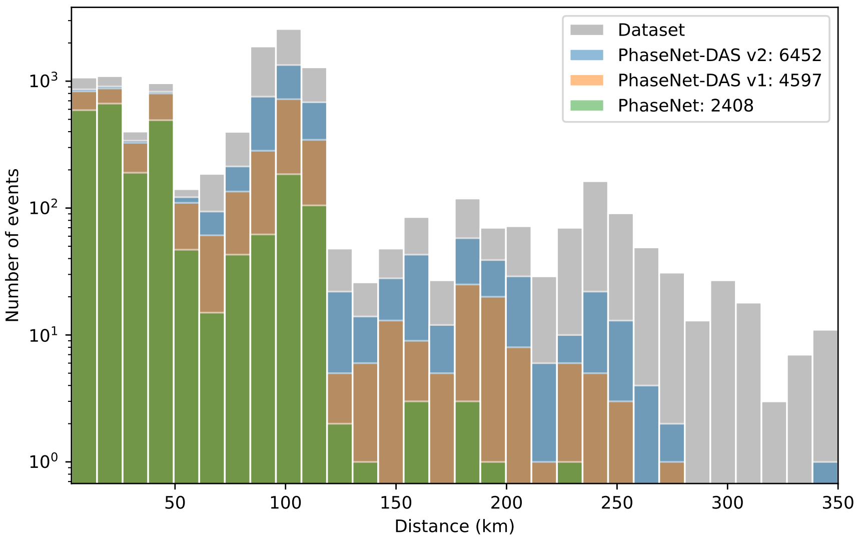

Fig. S3: Comparison of the number of earthquakes detected using the Mammoth north and Mammoth south cables.

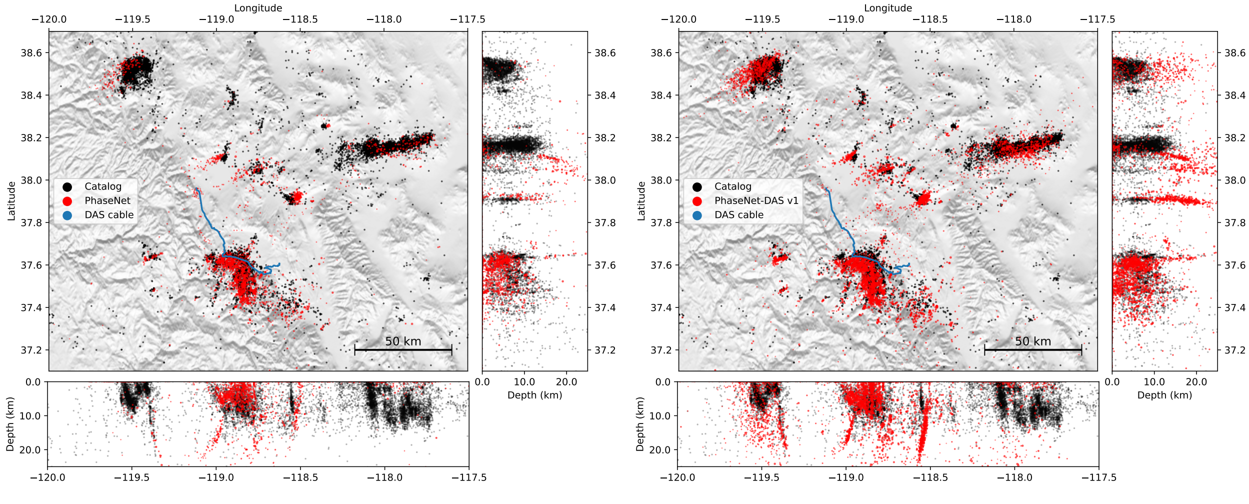

(a) (b)

Fig. S4: Earthquake locations determined by phase arrival-times from PhaseNet and PhaseNet-v1. The black dots are earthquakes in the standard earthquake catalogs. The red dots are earthquake determined by the DAS arrays and two methods: (a) PhaseNet and (b) PhaseNet-DAS v1. The results of PhaseNet-DAS v2 is shown in Figure 5.

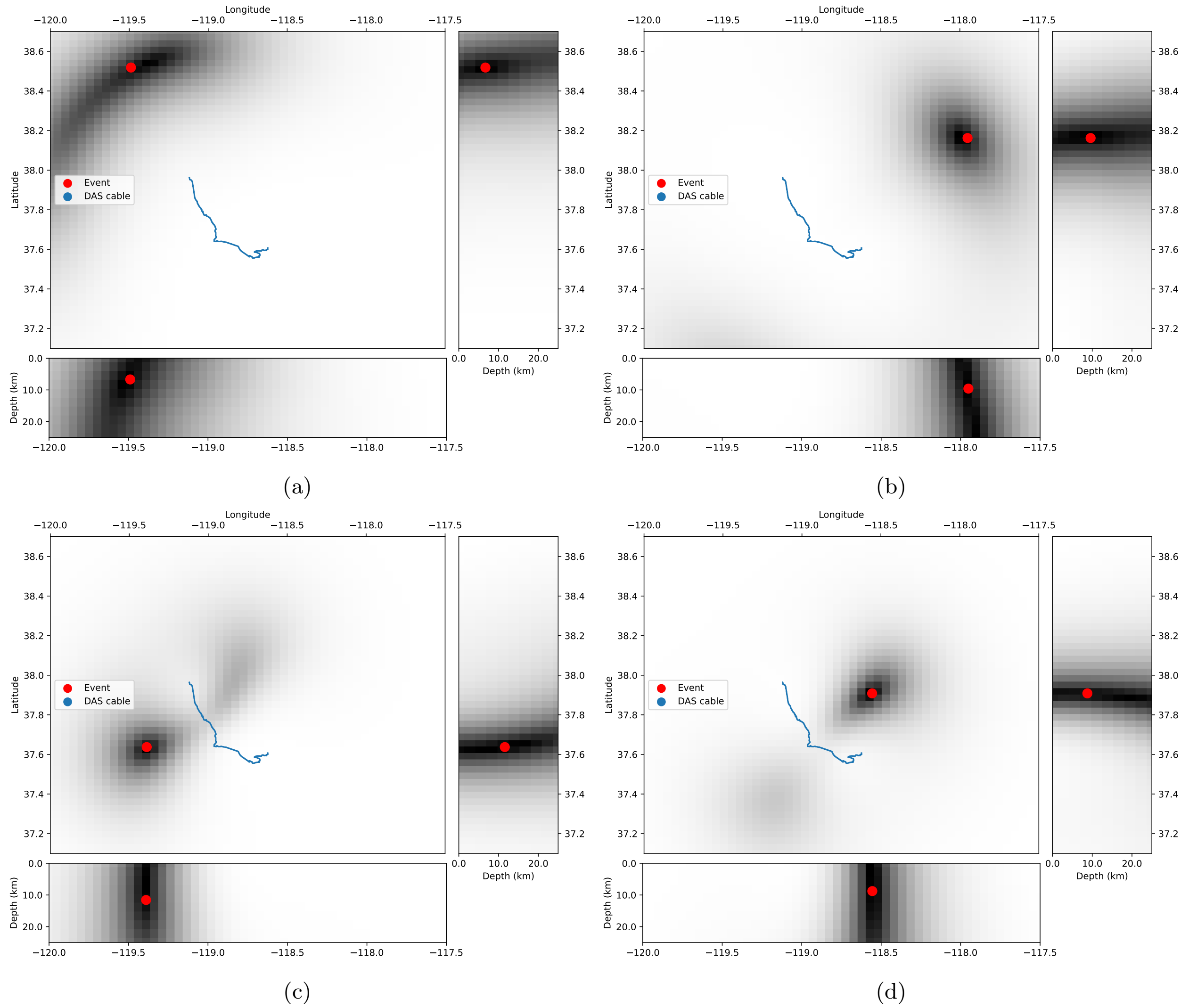

Fig. S5: Normalized loss surfaces of travel-time-based earthquake locations. The red dots are representative earthquake locations in Figure 5. Based on synthetic P and S arrival times, we apply a grid search to the latitude, longitude, and depth of earth locations and normalize the travel-time errors following $\frac{\exp -\frac{1}{N}\sum_N t_{ijk}}{\sum_{ijk}\exp -\frac{1}{N}\sum_N t_{ijk}}$, where t_{ijk} is the travel-time error for an earthquake location at grid (i, j, k) and N is the number of DAS channels. The loss surfaces show the location uncertainty due to limited azimuth coverage of the DAS cable shown in blue.

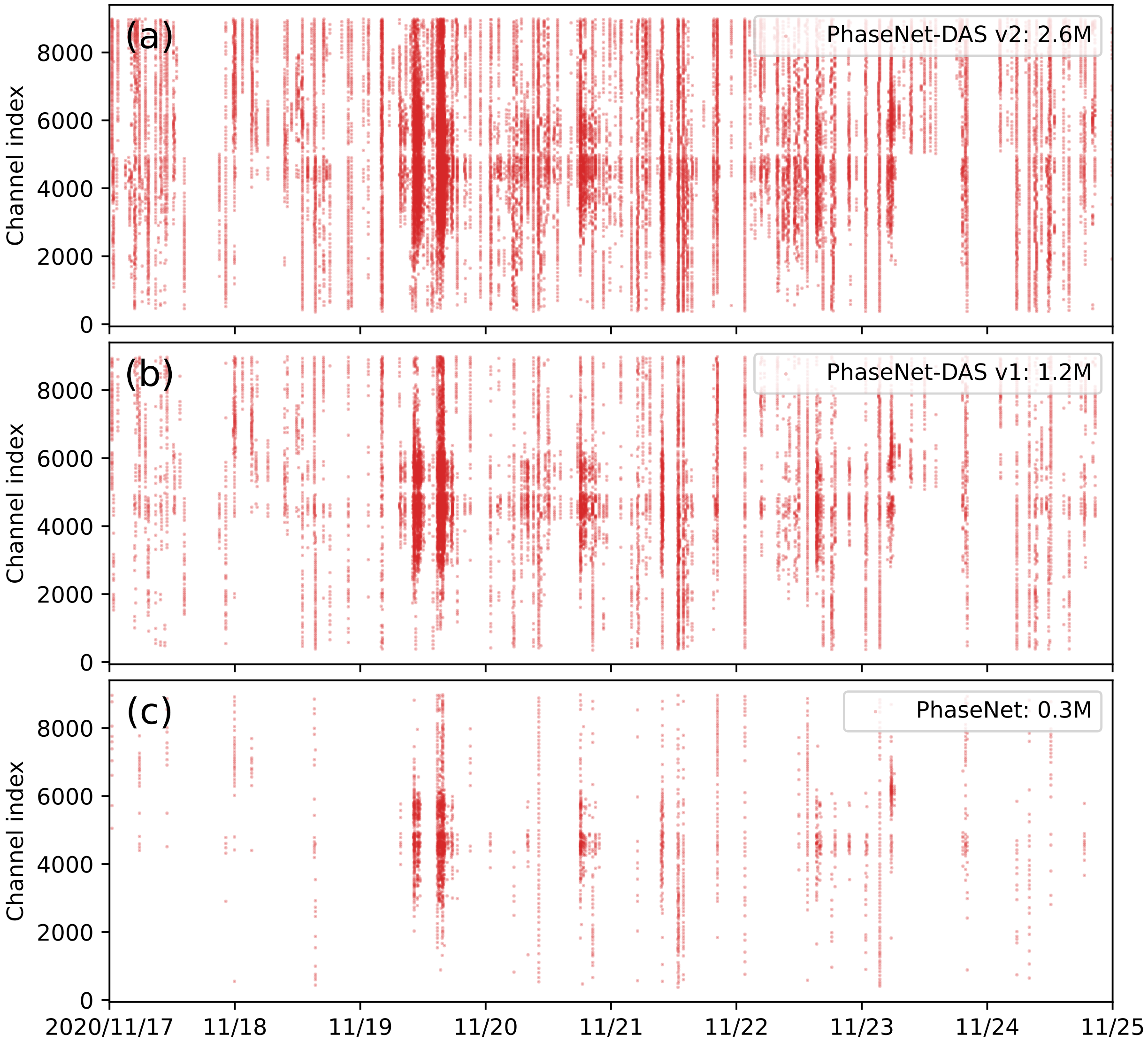

Fig. S6: Associated phase picks from continuous data: (a) PhaseNet-DAS v2, (b) PhaseNe-DAS v1, (c) PhaseNet. The channel indexes are organized from north to south along the DAS array. Each moveout across channels represents one group of picks from an earthquake. The histogram of associated events are shown in Figure S7.

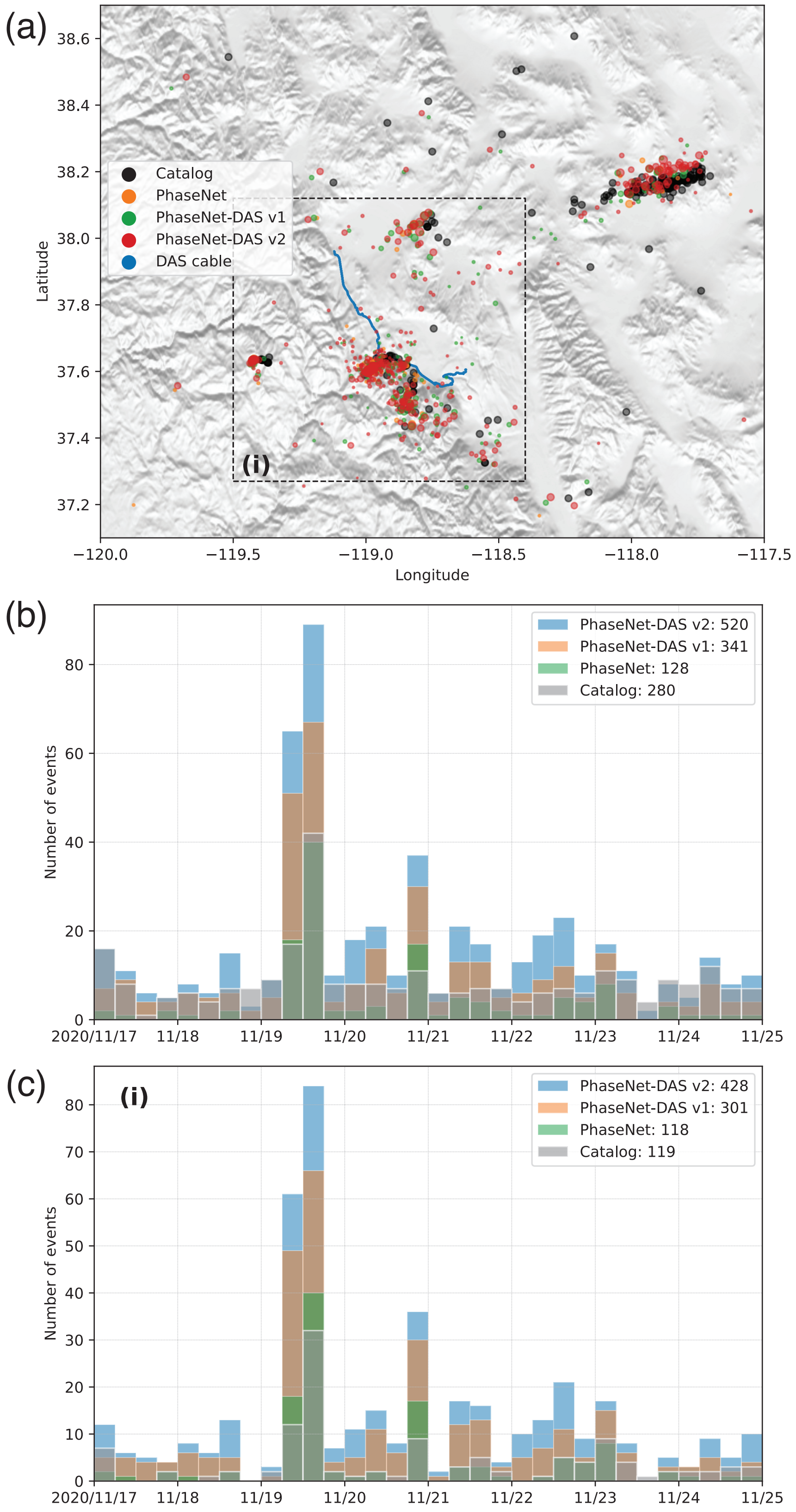

Fig. S7: Associated events from continuous data: (a) associated earthquake locations, (b) temporal distribution of event frequency, (c) event frequency within area (i) near the DAS cable.
